# Droplet Velocity in an Electrowetting on Dielectric Digital Microfluidic Device

**DOI:** 10.3390/mi7040071

**Published:** 2016-04-20

**Authors:** Mun Mun Nahar, Jagath B. Nikapitiya, Seung M. You, Hyejin Moon

**Affiliations:** 1 Department of Mechanical and Aerospace Engineering, The University of Texas at Arlington, Arlington, TX 76019, USA; munmun.nahar@mavs.uta.edu (M.M.N.); jagath.nikapitiya@gmail.com (J.B.N.); 2 Department of Mechanical Engineering, The University of Texas at Dallas, Richardson, TX 75080, USA; you@utdallas.edu

**Keywords:** electrowetting on dielectric (EWOD), digital microfluidics, droplet speed, switching time, velocity measurement, slender electrodes

## Abstract

In many electrowetting on dielectric (EWOD) based microfluidics devices, droplet actuation speed is a crucial performance-controlling parameter. Our present study aims to characterize and study droplet speed in a typical EWOD device. First, a practical droplet speed measurement method has been methodically demonstrated and some related velocity terms have been introduced. Next, influence of electrode shape on droplet speed has been studied and a new design to enhance droplet speed has been proposed and experimentally demonstrated. Instead of using square shaped electrodes, rectangular electrodes with smaller widths are used to actuate droplets. Additionally, different schemes of activating electrodes are studied and compared for the same applied voltage. The experiments show that a particular scheme of activating the array of rectangular electrodes enhances the droplet speed up to 100% in comparison to the droplet speed in a conventional device with square shaped electrodes.

## 1. Introduction

In recent years, significant efforts have been devoted to the development of droplet-based lab-on-a-chip devices of which advantages include being programmable and reconfigurable. Among various droplet flow based microsystems [1,2,3,4,5], electrowetting on dielectric (EWOD) digital microfluidics has many advantages, such as rapid switching response, no joule heating, no need for moving parts like pumps and valves, and, most importantly, low power requirements [6]. Basic droplet handling techniques—droplet dispensing, transporting, merging and splitting—can be done by sequentially activating and deactivating specific electrodes, which allows to address each droplet individually and to perform various unit processes, such as encapsulation [7], mixing [8], extraction [9,10] and separation [9] in lab-on-a chip devices. All of these applications are limited to mostly parallel plate EWOD device. Recently, droplets have been manipulated by AC voltages in microchannels for various applications, including a music jukebox [11,12,13], with more versatility.

Understanding of the dynamics of droplet motion in an EWOD device is crucial to design and build devices in various applications. To date, many researchers have experimentally and numerically investigated droplet dynamics in EWOD. The review article by Mugele and Baret [6] discussed approaches to understand the electrowetting theory applicable for low voltages. They analyzed the origin of electrostatic forces that reduce the apparent contact angle and induce droplet motion. They also briefly discussed droplet dynamics.

In EWOD, a series of electrodes are activated in a timed manner and a discrete droplet is actuated along these sequentially “fired” electrodes. The nature of the net electrostatic force on the droplet is not constant while a constant voltage is applied, because the actuation force depends on the dynamic shape of the droplet, which keeps changing due to deformation during transition from the grounded electrode to the activated one [6]. Therefore, in practical cases, the velocity field of a droplet varies with time during its transition. In order to fully understand EWOD actuation, we need to study a complex unsteady problem. In some lab-on-a-chip devices utilizing EWOD, droplet speed needs to be maximized to enhance performance [14,15]. For achieving maximum droplet speed, it is necessary to identify the parameters to favorably tune the basic forces of the system to indirectly control the speed. Some parameters, such as liquid surface energy [16,17], liquid viscosity [17], contact angle hysteresis [17], contact line friction [16,17], dimensions [18,19,20] and shape of electrodes or droplet [20,21,22], gap between electrodes [18,20] and the channel height [19] have been investigated numerically [18,19,20,21,22,23] and experimentally [22].

As already discussed, controlling droplet speed may become very crucial in many of the EWOD applications towards achieving optimum device performance. However, to the best of our knowledge, none of the studies presented specific guidelines to accurately characterize and measure droplet speed. For example, in References [18,19], the droplet velocity was measured in terms of the switching speed—the distance travelled by a droplet during one switching period. However, this measurement method bears serious errors unless the switching time is carefully controlled so that a droplet does not sit idly during a switching period. Therefore, a proper characterization of switching time is required to estimate droplet velocity accurately [24]. It becomes very important since, unlike droplet motion in microchannels, where the pressure drop is kept regular, droplet motion in EWOD device is very discrete, periodic, and unsteady in nature because of the on and off repetition of voltage. Therefore, the primary goal of this study was to establish a practical characterization method of droplet speed. The majority of previous studies on droplet velocities in an EWOD device have been focused on numerical studies and parameters that might have intrinsic restrictions in practical applications. A much lower number of experimental studies have been reported regarding droplet speed in an EWOD device. Since electrode design is a very convenient parameter to implement in any kind of practical application, we studied droplet velocity in an EWOD device with new electrode designs. In addition, we studied the electrode operation sequence effect on droplet velocity.

## 2. Methods

According to the rigid body model considered by Chatterjee *et al.* [25], and Bahadur and Garimella [21], both the droplet actuation force and the opposing forces during motion influence the droplet dynamics, which depend on the shape of the droplet base area (*i.e.*, contact area or footprint area). For example, the average actuation force for a droplet with a rectangular contact area will be greater than that of a droplet with a circular contact area. In the case of a dynamic droplet, the shape itself is affected by the deformation that the droplet undergoes, and eventually it affects droplet velocity [16,18]. We will later show, in this study, that droplet deformation can be controlled by designing and operating electrodes in a certain way. Beforehand, droplet deformation will be briefly explained.

### 2.1. Droplet Deformation

**Figure 1:**
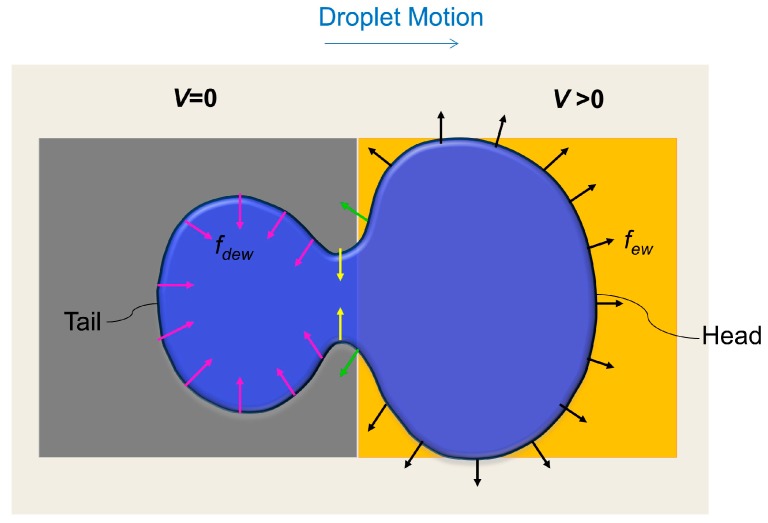
A droplet during transition from a grounded electrode to an activated one (Top view). Electrowetting force, *f*_ew_ (black arrows), and dewetting force, *f*_dew_ (pink arrows), on triple point contact line are shown. The green and yellow arrows show the dewetting and wetting forces adjacent to the junction of the activated and grounded electrodes.

According to Berthier *et al.* [26], an electrowetting force always acts normally to the droplet meniscus in an outward direction, whereas a dewetting force acts in an inward direction, which is also normal to the meniscus. Electrowetting and dewetting force per unit length of the meniscus can be expressed as follows:

*f*_ew_ = γcosθ(*V*) and *f*_dew_ = γcosθ(0) (1)

where γ is the liquid-gas interfacial tension, θ(*V*) is the apparent contact angle corresponding to an applied voltage *V*, and θ(0) is the apparent contact angle corresponding to a zero applied voltage. Figure 1 illustrates a dynamic droplet at the instant when the droplet partially wets an actuated (*V* > 0 applied) electrode and a grounded (*V* = 0) electrode. As shown in Figure 1, the electrowetting force on the leading meniscus tries to spread the droplet towards the electrode edges. At the same time, the dewetting force acting on the receding meniscus tries to pull the meniscus in an inward direction. Special attention should be paid to the interface between the actuated and grounded electrodes. On the grounded side of the interface, opposing dewetting forces squeeze part of the droplet inward (*i.e.*, yellow arrows in Figure 1). On the other hand, on the actuated side of the interface, electrowetting forces let the meniscus spread outward (*i.e.*, green arrows in Figure 1). As a result, a neck is formed at the interface and a head and a tail are formed in a dynamic droplet. If a very high voltage is applied, the velocity of wetting meniscus is very high and the droplet may split in two.

It is worth mentioning that the above explanation about deformation does not take into account several important factors, such as contact angle hysteresis, viscous, and shear effects. Our explanation of capillary line forces acting on the three phase contact line can only explain the deformation of the droplet footprint (base area of droplet on top and bottom surfaces), in terms of *x-y* curvature change. However, EWOD droplet motion is a three-dimensional phenomenon and to get the complete picture of deformation, it might be necessary to consider the *z*-directional curvature change, which might be affected by shear or contact angle hysteresis. However, since capillary number in the present study is less than one (Ca ~ 4 × 10^−4^ << 1), deformation is mainly governed by capillary forces, and viscous effects may be neglected. Therefore, a 2-D model can predict the droplet deformation well enough. Additionally, for our present purposes, we are more interested in exploiting the idea of manipulating the capillary forces to enhance droplet motion.

As we can see from Figure 1, if most of the meniscus remains on the activated electrode, there will not be any neck formation and droplet meniscus will be pulled outward, thus trying to overlap the activated electrode surface. Therefore, the droplet will manage to have an almost rectangular shape, similar to the shape of the activated electrode beneath it. According to the study by Bahadur and Garimella [21], this rectangular-shaped droplet can, theoretically, give the maximum droplet velocity.

While keeping the above discussion in mind, we can assume that electrode geometry can significantly affect droplet deformation. Therefore, in the current study, we tried to decrease the transition distance or the effective electrode width to reduce deformation. An array of slender electrodes was introduced for this purpose. In addition, different electrode operating schemes were tested to allow more of the droplet meniscus to have wetting forces, even in the beginning of the transition, to obtain a higher velocity. Note that, in conventional approaches with square electrodes, a droplet remains completely on a grounded surface, thus experiencing very small wetting forces at the beginning of the transition.

### 2.2. Electrode Design and Operation Sequence

**Figure 2:**
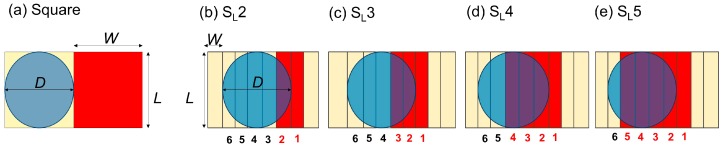
Schematic top view of: (**a**) Square electrode; (**b**)–(**e**) Slender electrodes with various electrode operation schemes. *L*, *W* and *D* denote electrode length, width and droplet diameter, respectively. Red areas represent the activated electrodes. The droplet moves from left to right in this figure.

In order to achieve a higher velocity for a given voltage, we proposed using an array of slender rectangular shaped electrodes. The dimension of each electrode is 2 mm × 0.4 mm (Figure 2b–e). Please note that width, *W*, is the dimension parallel to motion and length, *L*, is the vertical dimension of the electrode with respect to direction of motion. For the purpose of comparison, we also fabricated square electrodes with the same length and width (2 mm × 2 mm) (Figure 2a). Further, in the case of slender rectangular electrodes, the number of simultaneously activated electrodes was varied to create variation in the initial actuation force and also to expose the droplet interface to different amounts of electrowetting and dewetting forces at different locations in order to have different dynamic droplet shapes. Four different operation schemes were studied, as shown in Figure 2b–e. The first scheme is named as “S_L_2”. “S_L_” refers to slender electrodes and the “2” indicates two electrodes being activated simultaneously. In this scheme, during each switching time, two strips of slender electrodes are activated. At the beginning of each switching time period, the droplet keeps in contact with one activated and four non-activated electrodes. Thus, the total length of the activated electrode is two electrodes width, or 0.8 mm, and total transition distance is one electrode width, or 0.4 mm, during each switching time period. Other sequences can be explained and named in the same manner, where the total numbers of activated electrodes are varied but the transition distance is kept constant. In these cases, transition distance refers to the distance travelled during one switching time period.

### 2.3. Fabrication of EWOD Devices

All EWOD devices were fabricated in Nanofab at The University of Texas at Arlington. Indium tin oxide (ITO) electrodes (~100 nm) were patterned by wet etching of ITO after photolithography on an ITO coated glass substrate. Dielectric layer (SU-8, 5 μm) and hydrophobic layer (Teflon, 300 nm) were spin-coated and oven baked. The details of the fabrication steps can be found in Appendix A.

### 2.4. Test, Data Acquisition and Analysis

To test different designs of devices, a 400 nL water droplet of DI water was placed in a device. The gap between the top and bottom plates of the devices were kept at 100 μm for all devices. The droplet motion was recorded using a high-speed camera (Model: Miro M310, Vision Research; frame rate: 1000 fps; resolution: 512 × 480) and later analyzed using Phantom CineViewer software. Experimental videos are included in the Supplementary Materials. Details of the velocity measurements are described in the next section.

### 2.5. Characterization: Defining Droplet Velocity and Minimum Switching Time

Since the aim of present study is to propose a new electrode geometry to obtain faster droplet motion, it is necessary to have a clear definition of velocity that can be suitably measured in practical settings for comparison purposes. One definition found in other references is “switching speed” [18,22]. A switching speed (*v*_s_) is defined as the distance travelled during a switching period. One limitation of this definition is that *v*_s_ does not necessarily gives us the true speed during the transition because the droplet may actually complete its travel in a much shorter time than the imposed switching period and sit idly during the remaining time of the period until the next electrode is activated. This means that droplet’s true speed can be much faster than *v*_s_. Therefore, it is important to control the switching time correctly. If our interest is to achieve the maximum speed for a given set of parameters including voltage, we need to set the minimum switching time (*t*_min_) to activate electrodes so that the electrode next in the travel path of the droplet would be activated as soon as the droplet has completely moved onto the current electrode. In this way, droplet motion will be continuous without any significant interruption and *v*_s_ will become close to the true speed of the moving droplet.

**Figure 3:**
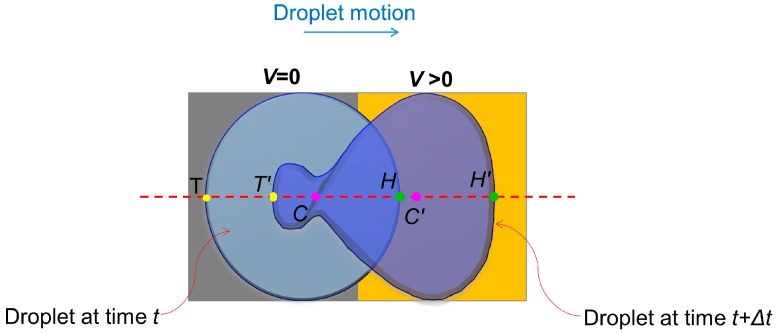
Measuring average velocity of a dynamic droplet. The droplet at left-hand side represents a droplet at any time instant, *t*, during transition from grounded electrode to activated electrode. The droplet at the right-hand side is the same droplet after time interval, Δ*t*. *T*, *T*′, *H*, *H*′, *C* and *C*′ denote three points designated as the droplet head, tail and centroid at two different time instants, respectively. The orange surface is the activated electrode. The red dashed line is the horizontal axis in the droplet.

In this study, droplet velocity was measured experimentally. A point on the droplet was first identified in a high-speed (1000 fps) camera images by analyzing frames. After every 50 frames for slender electrodes and 100 frames for square electrodes, the location of that particular point was identified and distance of the same point from the previous frame to the current frame was measured. Then, the distance was divided by the time interval between these two frames to obtain the velocity of the particular point. For a practical measurement of the average velocity of a droplet, three points on the horizontal axis of the droplet’s motion were considered. The front-most point on the droplet meniscus was designated as the droplet “head” (*H*), the back most point is the droplet “tail” (*T*), and the centroid of the droplet is (*C*) (Figure 3). To avoid rigorous calculations, the average of head, tail and centroid velocities was considered as a rough estimation of the droplet velocity (*v*_droplet_) of its horizontal motion:

The time-averaged droplet velocity (*v*_avg_) during the entire course of droplet travel will be:

(2) $$v_{\text{avg}}=\;\frac{1}{t^{*}}\;\int_{0}^{t}v_{\text{droplet}}\;\left(t\right)dt$$

where t* is the time to reach the destination.

## 3. Results

### 3.1. Minimum Switching Time (t_min_)

Since *t*_min_ is unique for each case, different switching times were tested for a droplet to move over a distance of 16 mm. The smallest switching time for which the droplet could reach the destination without any interruption was considered as *t*_min_. For switching times larger than *t*_min_, the droplet completes the motion earlier than the given time on each electrode, and the motion is interrupted in each period (Video S1). Whereas, if the switching times are smaller than *t*_min_, the droplet would stop permanently after traveling some distance (Videos S2 and S3). This is because the droplet cannot complete its transition over the current electrode and touch the next electrode within the switching time. The definition of switching speed using the *t*_min_ (*v*_s_ = *W*/*t*_min_) is close to the average droplet velocity (*v*_avg_). It has to be noted that the resolution of the switching time in our experiment was limited to 1 ms due to the facility’s capability. Therefore, it is probable that a droplet momentarily stops in each switching time period before it starts moving again to the next electrode, which results in a slightly longer *t*_min_ measurement than it should be. Thus, if we consider *v*_s_ to represent the droplet velocity, it can give a slightly lower value of the best achievable speed. For an applied voltage of 150 V_rms_, *t*_min_ for the square electrode device was close to 100 ms (Video S4). In the slender electrode array device, *t*_min_ were 10 ms (Video S5), 11 ms (Video S6), 11 ms (Video S7), and 10 ms (Video S8) for S_L_2, S_L_3, S_L_4, and S_L_5 schemes, respectively.

### 3.2. Droplet Velocity Measurement—Square Electrodes

**Figure 4:**
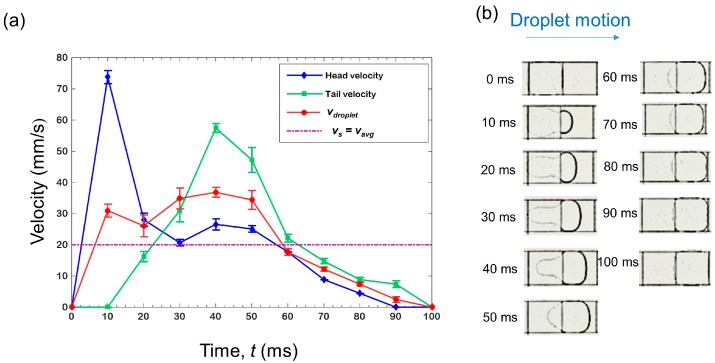
(**a**) Droplet velocity (*v*_droplet_) profile on a square electrode during one switching period. Velocities were calculated from three tests. Error bars reresent the standard deviation from the mean. Average velocity (*v*_avg_) and switching speed (*v*_s_) are equal and depicetd in purple dashed line. (**b**) Images of droplet shapes at different instants during *t*_min_ (=100 ms) on a square electrode.

Droplet velocity (*v*_droplet_) was measured as described in Section 2. Figure 4a shows the *v*_droplet_ profile of a droplet on square electrodes during *t*_min_ (≈100 ms) (Video S4). Droplet velocity gradually increases, reaches to the maximum value of 40 mm/s at about 40 ms and gradually decreases. The evolution of the droplet shape during *t*_min_ is shown in Figure 4b. In addition to *v*_droplet_, the head and the tail velocities of the droplet are overlaid in Figure 4a to highlight their differences, which caused significant droplet deformations. At the beginning, the droplet head accelerates and the front part of the droplet touches the activated electrode (from 0 to 10 ms). Simultaneously, the droplet deforms due to interplay between electrowetting and dewetting forces. In Figure 4b, at 10 ms, the droplet shows two distinct parts: A smaller half-circular leading portion and a larger circular receding portion. These two parts are joined at a narrow section near the junction of two electrodes. This narrow section is described as the “neck” in Section 2.1. In the neck region, the Laplace pressure should be very high due to increased curvature. This high pressure opposes the motion of the fluid on the grounded electrode. This trend continues until 30 ms. However, after a significant portion of the droplet has been transported over the activated electrode, the neck diminishes and, accordingly, droplet velocity increases again and reaches a maximum value at 40 ms. After 40 ms, most of the droplet is on the activated electrode and there is much less deformation. From this time onward, the droplet velocity decreases and it almost completes its transition at around 90 ms. This decrease in velocity at the latter part of its travel can be explained by the evolution of the electrowetting force as a function of the droplet footprint area over the activated electrode. The electrowetting force, as well as droplet velocity, gradually decreases after the droplet has reached at the middle of the activated electrode and vanishes when it covers the whole electrode. For a rigid circular droplet, the velocity profile, thus, takes a parabolic shape. The maxima of this parabola should be at the time when the droplet has reached half of the transition distance in the case where there is no dielectric material in the top plate [21]. Similarly, in our study, the droplet velocity reaches the maximum value of 38 mm/s at 40 ms, at which time the droplet has passed the halfway point of the electrode length. In Figure 4a, the *v*_droplet_ profile fluctuates slightly and it may be due to only considering three points in order to calculate *v*_droplet_. Having more points to average out the droplet velocity should give a smoother *v*_droplet_ profile. Note that *v*_s_ and *v*_avg_ are the same for the square electrodes case, and it is due to the fact that a droplet deforms and restores its shape again within one switching period.

### 3.3. Droplet Velocity Measurement—Slender Electrodes

**Figure 5:**
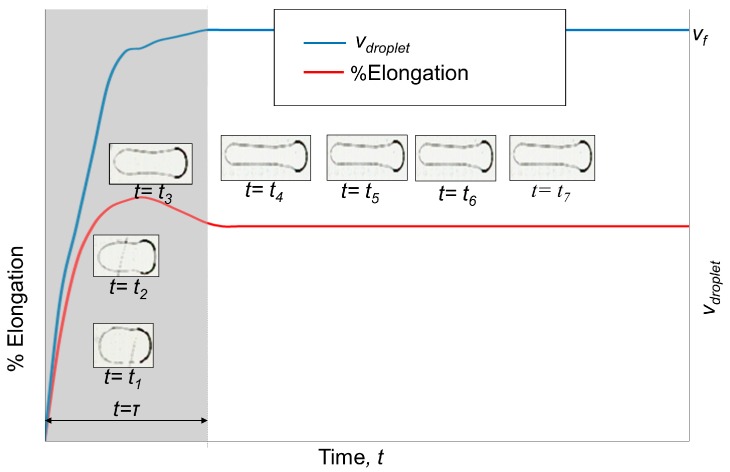
A representative droplet velocity (*v*_droplet_) profile and % elongation *vs.* time for slender electrode arrays in S_L_2 scheme. The shaded area represents the “transition region” and the remaining area of the plot represents “constant shape region”. The inset photos show the droplet profiles at different instants of those two regimes.

The droplet deformation patterns on slender electrodes are significantly different from those of the square electrodes case. Videos for all of the slender electrodes are available in the Supplementary Materials (Videos S1, S2 and S5–S8). Unlike the square electrodes case, a droplet does not restore its shape within one switching time, but continuously deforms while it transits over several electrodes until it reaches a certain amount of deformation. As a primitive indicator of deformation, the elongation of a droplet in the direction of motion was considered. By analyzing frames extracted from high-speed videos, the elongation in different frames was measured. The relative elongation, with respect to the initial dimension of the droplet (for S_L_2 scheme), was plotted in Figure 5. When the switching time is almost equal to *t*_min_ (Video S5), after the initial continuous deformation, the droplet stops deforming and continues to move in its final deformed shape. We call the initial distance having a continuous deformation as the “transition region”, and the later one, with no further deformation, the “constant shape region”. The time to reach the constant shape region is the transition time (τ). Droplet velocity, *v*_droplet_, sharply increases during the transition region and reaches a final value (final velocity, *v*_f_) once it arrives at the constant shape region and maintains it until the destination (Figure 5). As shown in the figure, a droplet in the S_L_2 scheme deforms very little while it goes through the constant shape region. This is true for all other schemes.

**Figure 6:**
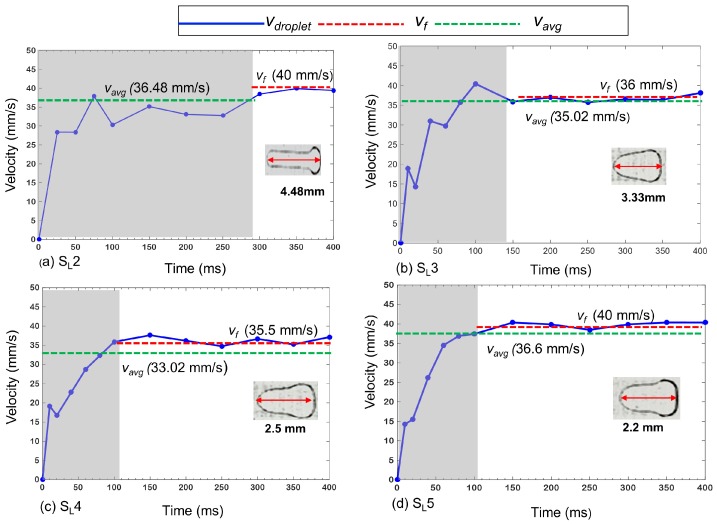
Experimental droplet velocity profiles for slender electrode arrays: (**a**) S_L_2, (**b**) S_L_3, (**c**) S_L_4 and (**d**) S_L_5. The insets show droplet shapes in the constant shape region. The shaded regions on the plots depict the transition regions. The red arrows in the insets represent elongated lengths of droplets in the direction of motion. Note that the footprint of the initial shape of the droplet of each case was 2 mm × 2 mm.

The plots of *v*_droplet_ in Figure 6 give an overview of the droplet motion for different schemes of the slender electrodes array. The inset photo of each plot is the droplet shape in the constant shape region. Firstly, *v*_droplet_ tends to be less in the transition region than in the constant shape region due to significant deformations in the transition region. In the present study, deformation is mainly controlled by operation schemes, as discussed in Section 2.2, which, in turn, controls the amount of actuation force. Scheme S_L_2 provides the smallest part of the droplet circumference with the wetting force compared to the other schemes, and it is the opposite for S_L_5. Hence, S_L_2 has the greatest and S_L_5 has the least elongation. Interestingly, S_L_2 has the longest transition time, τ of ~300 ms. (Figure 6a). For the S_L_2 scheme, *v*_avg_ is 36.48 mm/s, while its final velocity (*v*_f_) is 40 mm/s. If the droplet moves a much longer distance in the constant shape region, so that t* >> τ, *v*_avg_ will be as high as the final velocity (=40 mm/s), which is almost twice that of *v*_avg_ of the square electrodes case. For S_L_3 (Figure 6b), the elongation and τ are smaller than those of S_L_2. The *v*_avg_ is 35.02 mm/s and the *v*_f_ is 36 mm/s. For S_L_4, τ is almost same as that of S_L_3 because the elongation of those two schemes is similar (Figure 6c). Although S_L_4 has the same *v*_f_ as that of S_L_3, a lower velocity during τ limits its *v*_avg_ over the 400-ms period to 33.02 mm/s. The highest *v*_avg_ of 36.60 mm/s is observed in S_L_5 (Figure 6d). Similar to S_L_2, the *v*_f_ of S_L_5 is about 40 mm/s, but since the τ in the case of S_L_5 is smaller, its *v*_avg_ over the 400 ms is larger (36.6 mm/s). It is noted that the elongation in S_L_5 is the smallest. The final velocities, *v*_f_ for all of the cases, match very well with the switching velocity, *v*_s_. Although we assumed that the droplet did not deform in the latter part of the transition, in fact, there was a minute instantaneous deformation, which is random in nature, depending on the local variations in the surface conditions. We neglected it as a significant factor to control droplet deformation. Table 1 provides a summary of the experimental results for the square, as well as four different cases of slender, electrodes.

## 4. Discussion

**Figure A1:**
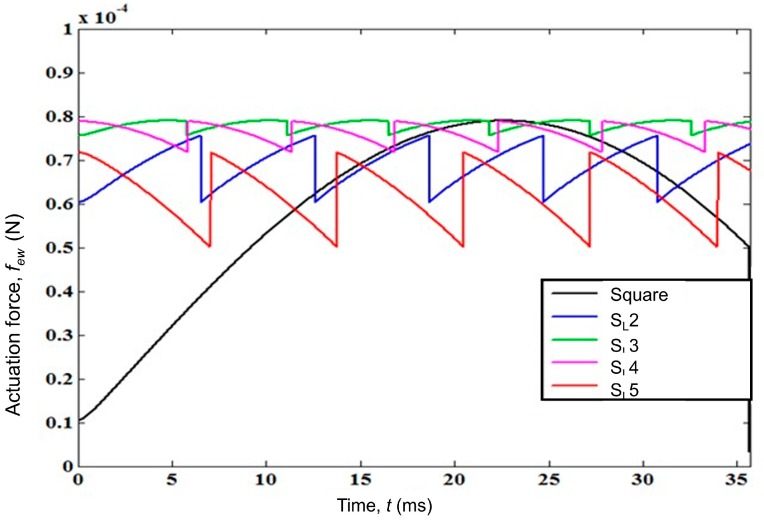
Actuation force (*f*_ew_) with time on a circular droplet for various electrode operation schemes (rigid body model).

To explain the reasons for getting a higher *v*_avg_ for slender electrodes than for square electrodes, the first assumption, made in Section 2.1, can be recalled. We know that the actuation force in EWOD is a function of transition distance and the maximum actuation force that can be achieved by operating the electrodes in a certain way, as we did in case of the slender electrodes. EWOD actuation force will vary by droplet shape because of the different gradients in the overlapped area (dA(*x*)/d*x*), which is generally a function of transition distance, unless a droplet is in a square shape [21]. Using MATLAB (MathWorks, Natick, MA, USA) and the theoretical formulations based on a rigid body model [21], we calculated the actuation force for a circular droplet for all of the experimental cases. All deformations were neglected, and the droplet was considered to behave like a rigid body. The droplet volume was the same for each case, and the droplet diameter was considered to be slightly larger than the electrode length. For the total actuation force calculation, we used an area integral in MATLAB during a transition time of 35.67 ms, for which a droplet travels 2 mm in a square electrodes device. The force plots have been provided in Appendix B (Figure A1). In the same period of time, droplets for other sequences experience different travelling distances. For example, in S_L_5, a droplet completes more than five transitions, equivalent to more than 2 mm (5 × 0.4 mm). Then, by assuming the time averaged actuation force, we could roughly compare the actuation forces of different sequences. This is tabulated in Table 2.

**Figure 7:**
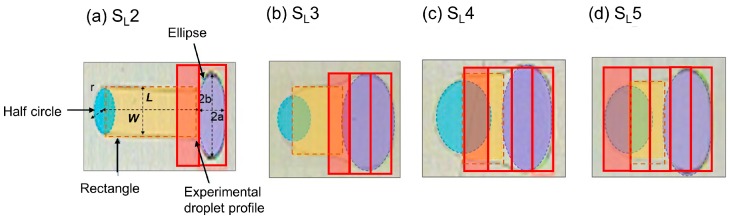
Approximate droplet shapes used for theoretical calculations of droplet motion on slender electrode arrays (**a**–**d**). For each sequence droplet footprint area consists of an elliptical front part (shaded in violet), rectangular middle part (shaded in light orange) and half circular back part (shaded in sky blue). Letters a and b denote the major and minor axis of the ellipse, *L* and *W* denote length and width of the rectangle, and r is the radius of the half circular part. The red outlines show the activated electrodes and the shaded portion shows the transition distance.

As seen in Table 2, all of the sequences of slender electrodes show a higher time averaged actuation force in the given time period than that of the square electrode case. When the droplet starts to move, it has a different overlapped area for the square, and also different schemes for the slender electrodes. Compared to the square electrode case, the initial actuation force for slender electrodes is much higher. In addition, since it transits only 0.4 mm (one electrode strip distance) during each transition, the overlapped area of the droplet with the activated electrode changes very little during this time, the actuation force also changes very little. If the droplet moves a 2 mm distance, it will have repetitive actuation force profiles corresponding to each switching time period, which happens several times, as opposed to the parabolic profile of square electrodes (Figure A1). When we calculate the time averaged actuation force for both cases, in general, we will always get a higher actuation force, as well as higher average velocity, for slender electrodes, which we derived from the experiments. One limitation of this theoretical calculation is that *t*_min_ for square electrodes from this modeling (=35.67 ms) is almost three times shorter than *t*_min_ from the experiment (≈100 ms), which indicates an overestimated actuation force or underestimated opposing forces that we predicted using this model. This difference is due to the fact that the droplet deforms significantly during one switching time in the square electrode case, which is neglected in the rigid body model. On the other hand, it is confirmed that droplet deformation is negligible within the constant shape region of S_L_ schemes, although they are not necessary circular or exactly square-shaped. Therefore, if we can update the shape of the droplet in the rigid body model from the circular shape to the final shape of the droplet in the constant shape region of the slender electrodes, the rigid body model may result in closer predictions to the experimental results. Thus, we modified the rigid body model by taking approximate droplet shapes for the slender electrodes cases, as shown in Figure 7, where we assumed the droplet base area to be composed of a combination of elliptical, rectangular, and half circular regions, as opposed to being completely circular.

With these updated droplet shapes, actuation force, average velocity (*v*_avg_) and minimum switching time (*t*_min_) were calculated for the slender electrodes using the rigid body model. The estimated switching times were 9.94 ms, 10.257 ms, 11.33 ms and 9.98 ms, for S_L_2, S_L_3, S_L_4, and S_L_5, respectively. The estimations using the rigid body model are very close to our experimental results, as shown in Table 3. We also confirmed the validity of the rigid body model by simulating velocity and pressure field using COMSOL Multiphysics software (COMSOL, Stockholm, Sweden) for square electrode case and S_L_5 scheme of slender electrodes. As shown in Appendix C, unlike in square electrode, the uniform and unidirectional velocity field in S_L_5 scheme justifies the assumption the droplet to be a rigid body. Thus, we can safely use the rigid body model results to explain higher velocities for different schemes of slender electrodes.

**Figure 8:**
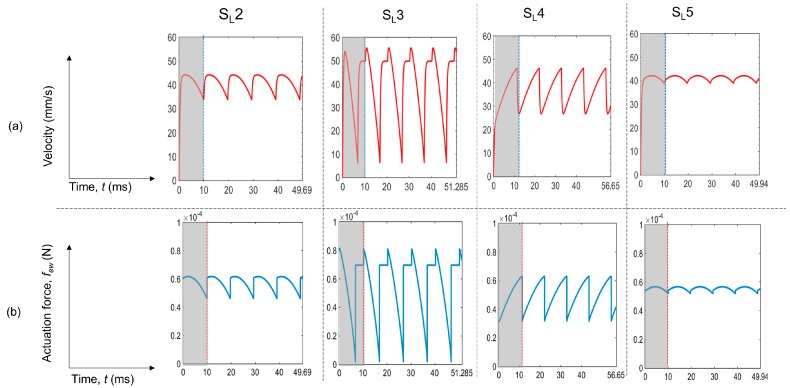
Theoretical (**a**) velocity profiles and (**b**) actuation force *vs.* time, for all of the cases of slender electrodes during the transition over a 2-mm distance. The shaded regions correspond to one switching period for each case.

Since the theoretical calculations give an almost similar *t*_min_, the velocity results should also be similar. As we can see in Figure 8a, the average velocity profiles for all of the cases are different depending on the shape of the contact area of the droplet with electrode. The time averaged velocity, *v*_avg_ calculated from the velocity plots in Figure 8a shows a very close match to the final velocities (*v*_f_) observed in the experiments (Table 3). During the transport of the droplet over a distance of 2 mm, the theoretical time averaged velocity for S_L_5 and S_L_2 is at a maximum, which is 41 mm/s compared to an average velocity of 40 mm/s for S_L_3 and 36.5 mm/s for S_L_4. In our experiments, we also get the maximum average velocities for sequences, S_L_2 and S_L_5 with the velocity averaging to about 40 mm/s in the constant deformation regions. For the other two sequences, the average is about 36 mm/s once the droplet travels beyond the constant shape region. Thus, after modifying the droplet shapes, the rigid body model can predict the results acceptably for the slender electrodes. The minute deviation from the experimental results is due to the difference between the approximated droplet shape from the actual droplet shape and also to the slight changes in shape during the motion in each switching period in the experiments that was neglected for simplicity in the theoretical calculation.

Next, the time averaged actuation force is calculated and shown in Figure 8b, and is also tabulated in Table 3. In general, the time averaged actuation force is higher for the sequence giving a higher velocity, except for S_L_5, which gives less actuation force than S_L_2 and S_L_3 but has a higher velocity than the two. This may be due to the fact that there might be some errors in the theoretical calculation resulting from both instantaneous deformations, which we neglected, and also inaccuracy in shape estimation during modeling. One observation is that the actuation force profiles are similar to the velocity profiles in Figure 8a. This means that if the droplet shape is constant or the deformation is much less, droplet velocity is mainly governed by the actuation force, which has been previously found in the literature [16].

## 5. Conclusions

In this study, a new design of electrode geometry has been proposed, and it has been demonstrated that, compared to the conventional square shaped electrodes, using slender electrodes can give almost 100% enhancement in velocity. The result has been validated and explained using theoretical modeling. Experimental protocols regarding the control of switching time for gaining an optimum velocity has been discussed. Additionally, a more practical definition for measuring droplet velocity has been presented.

## Figures and Tables

**Table 1:** Summary of experimental results.

Electrode Type	Minimum Switching Time, *t*_min_ (ms)	% Elongation	Transition Time, τ (ms)	Final Velocity, *v*_f_ (mm/s)	Average Veloctiy, *v*_avg_ (mm/s)
Square	97	-	-	-	20
S_L_2	10	124	300	40	36.48
S_L_3	11	25	150	36	35.02
S_L_4	11	16.5	100	35.5	33.02
S_L_5	10	10	100	40	36.60

**Table 2:** Percentage increase in actuation force of slender electrode sequences with respect to a square electrode.

Operation Sequence	% Increase in Actuation Force
S_L_2	16
S_L_3	32
S_L_4	29
S_L_5	6

**Table 3:** Comparison between theoretical calculation (rigid body model) and experimental results for slender electrodes.

Operation Sequence	Actuation Force (μN)	Theoretical Average Velocity, *v*_avg_ (mm/s)	Experimental Final Velocity, *v*_f_ (mm/s)	Theoretical *t*_min_ (ms)	Experimental *t*_min_ (ms)
S_L_2	0.575	41	40	9.94	10
S_L_3	0.563	40	36	10.257	11
S_L_4	0.4966	36.5	35.5	11.33	11
S_L_5	0.546	41	40	9.98	10

**Figure A2:**
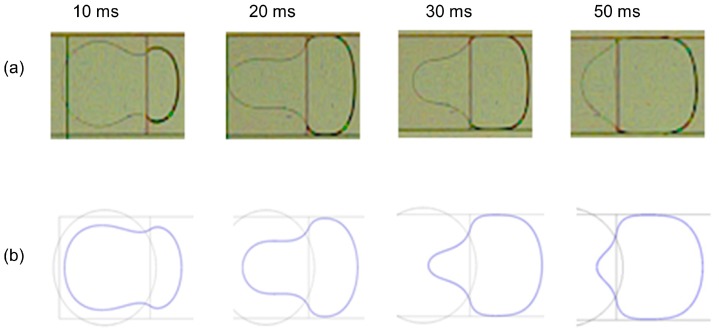
Droplet interfaces on square electrodes at different time intervals. (**a**) Images from the experiment; (**b**) Droplet free surface profiles from simulation (in blue).

**Figure A3:**
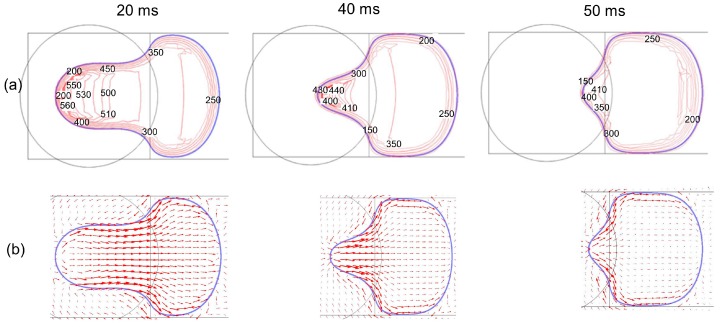
(**a**) Pressure distribution inside droplet on square electrode from simulation and (pressure contours in MPa); (**b**) velocity field inside droplet from simulation. Interface shown in blue and initial droplet shape is shown in black.

**Figure A4:**
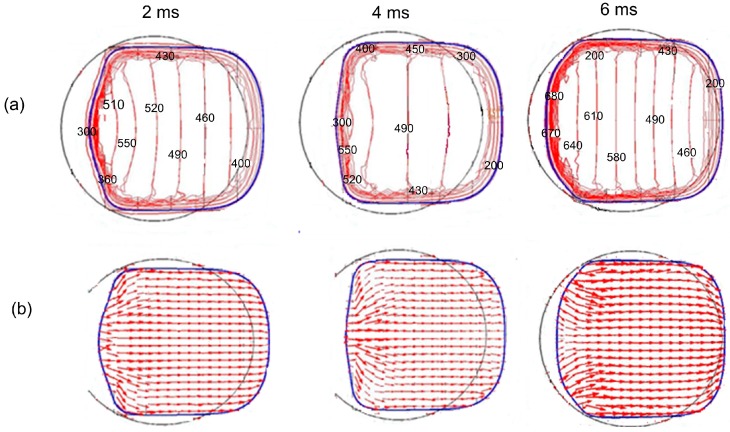
(**a**) Pressure distribution inside droplet on slender electrodes (S_L_5) from simulation (pressure contours in MPa); (**b**) velocity field inside droplet from simulation (interface shown in blue and initial droplet shape is shown in black).

## Supplementary Materials

The following are available online at http://www.mdpi.com/2072-666X/7/4/71/s1. Video S1: Experimental video for S_L_2 with 11 ms switching time; Video S2: Experimental video for S_L_2 with 9 ms (*t*_min_) switching time; Video S3: Experimental video for Square electrodes with 95 ms switching time; Video S4: Square electrodes with 100 ms (*t*_min_) switching time ; Video S5: Experimental video for S_L_2 with 10 ms (*t*_min_) switching time; Video S6: Experimental video for S_L_3 with 11 ms (*t*_min_) switching time; Video S7: Experimental video for S_L_4 with 11 ms (*t*_min_) switching time ; Video S8: Experimental video for S_L_5 with 10 ms (*t*_min_) switching time.

## Appendix A

An ITO coated glass substrate was used. The first step of the process was to clean the wafer thoroughly with non-halogenated hydrocarbons: Acetone, methanol, iIsopropanol and then rinsing with DI water following a dehydration process at 150 °C for 2 min. First, a thin layer of HMDS was spin coated using the following recipe: Spin speed was ramped up to 500 rpm, 100 rpm/s for 5 s; ramping with 900 rpm/s to 4000 rpm for 30 s, which provided a very thin and uniform adhesive layer of HMDS between the glass substrate and the photoresist, which will be deposited next. The ITO coated glass substrate with the coated HMDS layer was baked at 150 °C for 1.5 min for hardening.

Then, a positive PR (Microchem S1813, MicroChem Corp, Newton, MA, USA) of 1.2 μm uniform thickness was spin coated on the wafer using the following recipe; spin speed was ramped up to 500 rpm by 100 rpm/s for 5 s; ramping with 900 rpm/s to 3000 rpm for 30 s. The glass substrate with the coated PR layer was baked to harden the PR at 115 °C for 1 min. After performing Photo Lithography with a light dose of 140 mJ/cm^2^ on the Backside-Aligner (OAI 806MBA), the wafer was soft baked at 110 °C for 1 min. After that, it was developed in a developer (Microchem, MF-319) and dehydrated at 115 °C for 2 min. After developing, the electrode pattern onto the PR layer was observed under the microscope for accuracy. The wafer was then agitated in a mixture of Hydrochloric (HCL) acid, Nitric (HNO_3_) acid and DI water (H_2_O) (wt %- 20% HCl, 5% HNO_3_, 75% H_2_O or vol %- 8:1:15, HCl:HNO_3_:H_2_O) for 2.5 min at 55 °C to etch the ITO layer. After PR stripping (Remover 1165, Microchem), the wafer was dehydrated at 150 °C for 2 min.

After that, a layer of dielectric (SU-8-5, Microchem) was spin coated on the wafer with following recipe; spin speed was ramped up to 500 rpm with 100 rpm/s for 5 s; ramping by 900 rpm/s to 2000 rpm for 30 s) resulting in a 5 μm thick uniform dielectric layer. The ITO coated substrate with the coated dielectric layer was then baked to harden the layer at 65 °C for 1 min and then 95 °C for 3 min. Photo Lithography was done for this dielectric layer coated glass substrate with a light dose of 140 mJ/cm^2^ on the Backside-Aligner (OAI 806MBA). The glass substrate was soft baked at 65 °C for 1 min, 95 °C for 1 min and also hard backed at 150 °C for 5 min. To provide hydrophobicity, a 300 nm thick uniform Teflon layer was spin coated on the dielectric coated glass surface with the following recipe: spin speed was ramped up to 1000 rpm by 300 rpm/s for 30 s. To increase surface uniformity, annealing was done at 150 °C for few hours. Finally, an ITO coated cover plate was first cleaned thoroughly with non-halogenated hydrocarbons: Acetone, methanol, isopropanol and then rinsed with DI water. The cover plate was then dehydrated at 150 °C for 2 min. A layer of Teflon with a 300-nm thickness was spin coated on the cover plate to provide hydrophobicity and annealed at 150 °C for few hours to increase the surface uniformity. Using a pipette, a drop of DI water (~0.4 μL) was dispensed onto the reservoir and the top plate was positioned to provide a uniform spacer gap between the bottom plate and the cover plate [27].

## Appendix B

The force plots for different electrode operation schemes are shown in Figure A1.

## Appendix C

To explain our experimental results from analyzing the pressure and velocity distribution inside a dynamic droplet, we solved the three-dimensional Navier-Stokes equation using COMSOL Multiphysics software for the square electrode case and S_L_5 for the slender electrodes. Although we did not consider contact angle hysteresis and contact line friction, good agreement between the numerical model and the experiment has been obtained. Figure A2 shows the droplet profiles on square electrodes derived from the simulation at different time intervals and are compared with the profiles from the experimental images at the same time instances.

Figure A3 and Figure A4 show the velocity and pressure field for the square electrodes and the S_L_5 of slender electrodes, respectively. As shown in Figure A3, most of the droplet deformation occurs during the first 50 ms in case of square electrode. In the next 50 ms of the switching time, the droplet restores its original shape at a very slow rate. Therefore, we are particularly interested in the first half of the switching time. Snapshots from numerical simulation show the pressure and velocity distributions at various time instants. At 10 ms, at droplet front part, especially near the neck region, an abrupt change in *x*-*y* curvature creates steep pressure gradients which give higher fluid velocity at that portion. At 20 ms, the trailing part of the droplet has higher pressure gradients, as well as higher velocity. This is again resulted from sharp curvature and smaller radius of curvature. At 50 ms, the trailing part of the droplet becomes smaller with a smaller radius of curvature. At this time, also, this portion of the droplet has a higher velocity. For the slender electrodes case, with S_L_5, as shown in Figure A4, the droplet remains almost square-shaped and the pressure gradients are uniformly distributed and have become parallel to each other and perpendicular to the direction of droplet motion. Because the droplet shapes are very different in both of the cases in our study, the local *x*-*y* curvatures are different, thus creating very different pressure profiles and velocity fields inside the droplet motion.
